# Gene expression patterns that predict sensitivity to epidermal growth factor receptor tyrosine kinase inhibitors in lung cancer cell lines and human lung tumors

**DOI:** 10.1186/1471-2164-7-289

**Published:** 2006-11-10

**Authors:** Justin M Balko, Anil Potti, Christopher Saunders, Arnold Stromberg, Eric B Haura, Esther P Black

**Affiliations:** 1 Department of Pharmaceutical Sciences, University of Kentucky, Lexington, KY 40536-0082, USA; 2 Institute for Genome Sciences and Policy, Duke University, Durham, NC 27708, USA; 3 Department of Statistics, University of Kentucky, Lexington, KY 40506-0027 USA; 4 Thoracic Oncology program, The H. Lee Moffitt Cancer Center & Research Institute, Tampa, FL 33612, USA

## Abstract

**Background:**

Increased focus surrounds identifying patients with advanced non-small cell lung cancer (NSCLC) who will benefit from treatment with epidermal growth factor receptor (EGFR) tyrosine kinase inhibitors (TKI). EGFR mutation, gene copy number, coexpression of ErbB proteins and ligands, and epithelial to mesenchymal transition markers all correlate with EGFR TKI sensitivity, and while prediction of sensitivity using any one of the markers does identify responders, individual markers do not encompass all potential responders due to high levels of inter-patient and inter-tumor variability. We hypothesized that a multivariate predictor of EGFR TKI sensitivity based on gene expression data would offer a clinically useful method of accounting for the increased variability inherent in predicting response to EGFR TKI and for elucidation of mechanisms of aberrant EGFR signalling. Furthermore, we anticipated that this methodology would result in improved predictions compared to single parameters alone both *in vitro *and *in vivo*.

**Results:**

Gene expression data derived from cell lines that demonstrate differential sensitivity to EGFR TKI, such as erlotinib, were used to generate models for *a priori *prediction of response. The gene expression signature of EGFR TKI sensitivity displays significant biological relevance in lung cancer biology in that pertinent signalling molecules and downstream effector molecules are present in the signature. Diagonal linear discriminant analysis using this gene signature was highly effective in classifying out-of-sample cancer cell lines by sensitivity to EGFR inhibition, and was more accurate than classifying by mutational status alone. Using the same predictor, we classified human lung adenocarcinomas and captured the majority of tumors with high levels of EGFR activation as well as those harbouring activating mutations in the kinase domain. We have demonstrated that predictive models of EGFR TKI sensitivity can classify both out-of-sample cell lines and lung adenocarcinomas.

**Conclusion:**

These data suggest that multivariate predictors of response to EGFR TKI have potential for clinical use and likely provide a robust and accurate predictor of EGFR TKI sensitivity that is not achieved with single biomarkers or clinical characteristics in non-small cell lung cancers.

## Background

Small molecule tyrosine kinase inhibitors (TKI) of the epidermal growth factor receptor (EGFR) can induce both tumor regression and disease stabilization when used as second line therapy in patients with advanced non-small cell lung cancer (NSCLC) [1-3]. Mutations in the tyrosine kinase domain of EGFR were observed in patients that responded to EGFR TKIs. Cell lines harboring mutated EGFR are dependent on EGFR for survival since inhibition of EGFR using TKIs, monoclonal antibody C225 or RNAi knockdown results in apoptosis [4-8].

While substantial data now exists that mutations in the tyrosine kinase domain of EGFR are associated with increased sensitivity to EGFR TKI, mutation in EGFR was not found to correlate with response to erlotinib in the BR21 trial [9]. More recent reports have suggested that increased EGFR gene copy number, co-expression of other ErbB receptors and ligands, and epithelial to mesenchymal markers are important in determining sensitivity to EGFR TKI [10-13]. There are conflicting reports about the role of RAS mutation and subsequent signalling in response to EGFR TKI [2,10,12]. In addition, identifying patients who may clinically benefit from EGFR TKI other than through overt tumor response remains unclear. Importantly, tumor regression has been observed with these agents in patients that did not have identifiable EGFR mutations, suggesting other mechanisms, such as activation of parallel signalling pathways, underlie responsiveness to these agents [8,14-16]. Therefore, the clinical decision on how best to choose patients for EGFR TKI remains an important and ongoing dilemma.

Development of molecular profiles as predictive measures of outcome or response to therapy has increased significantly since the advent of large-scale genomic and proteomic approaches for classification of cancers [17]. Microarray technology allows for interrogation of large numbers of genes that encompass variability found in biological conditions. However, methods of data analysis and modelling are hampered by the data itself in that it involves significantly more data points than experiments primarily due to the cost associated with performing many replicates [18,19]. Thus, building predictive profiles of clinical outcome or therapeutic response in non-small cell lung cancers using large-scale genomic data is a daunting process, but may be necessary for improving patient-targeted therapy.

We developed a novel methodology using both bioinformatics approaches and supervised learning methods to model sensitivity to EGFR inhibitors with gene expression data from lung cancer cell lines. Cell lines were chosen as tumor surrogates for ease of handling, the ability to assay EGFR and downstream signalling events by biochemical methods, and the capacity to test inhibitors in a controlled environment. The predictive models were subjected to extensive leave-one(or a group)-out cross-validation as well as out-of-sample validation using gene expression data from additional cell lines and human tumors. The predictive models described here are both robust and accurate predictors of response which exceed the capacity of single parameters alone in NSCLC cell lines. Our data suggest that this finding may be translated to *in vivo *tumors with similar value.

## Results

### Identification of sensitive and resistant cancer cell lines

**Figure 1:**
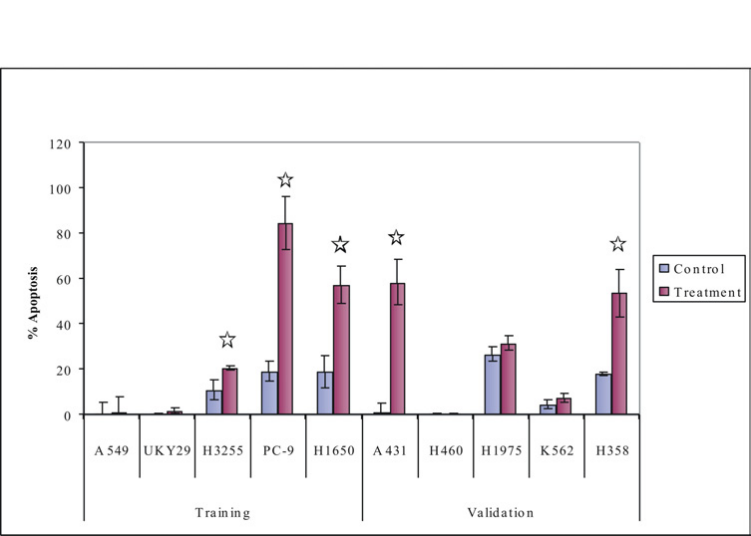
**Sensitivity to erlotinib in cell lines**. Sensitivity to EGFR tyrosine kinase inhibitors was determined by treating cells with 1 μM erlotinib for 72 hours under serum-starved conditions. Apoptosis was assessed by integration of the sub-G_1 _peak and compared to cells treated with equal volume of vehicle (DMSO). Experiments were repeated in triplicate with error bars representing standard deviation. : denotes statistical significance (p < 0.05, two sided t-test for unequal variances).

Using lung cancer cell lines as tumor surrogates, we sought to find gene expression patterns that can predict the sensitivity to EGFR tyrosine kinase inhibitors. Published data, and our own, demonstrate that lung cancer cell lines are differentially sensitive to EGFR inhibitors, likely reflecting dependency upon EGFR or related signalling pathways [20]. We identified lung adenocarcinoma cell lines sensitive to a representative EGFR TKI, erlotinib, by DNA content analysis using propidium iodide staining. Apoptosis was assayed by quantifying the sub-G1 peak following propidium iodide staining and FACS analysis in cells treated with 1 μM erlotinib for 72 hours or DMSO control (Figure 1). Several cell lines tested were sensitive to treatment with 1 μM erlotinib and these data are consistent with the findings of others [13,20]. We selected the A549 and UKY-29 cell lines for the drug-resistant training group, and the H1650, H3255, and PC-9 cell lines for the drug-sensitive training group.

### Sequence analysis of EGFR and K-Ras genes

Since EGFR and K-Ras mutational status are thought to correlate with sensitivity and resistance to EGFR TKIs, respectively [21], we characterized the mutational status of EGFR and K-Ras in the cell lines. The status of K-Ras and EGFR has been previously determined in all of the cell lines used, except lung adenocarcinoma cell line UKY-29, isolated at the University of Kentucky. We performed direct DNA sequencing to identify mutations in EGFR exons 18–21 as well as K-Ras exons 1 and 2 in the UKY-29 cells as previously described [22,23]. The UKY-29 cells are wildtype for EGFR and harbour a mutation (G61H) in exon 2 of K-Ras which has been observed in other NSCLC tumors and cell lines. A summary of the cell line data is shown in Table 1.

### Microarray analysis and feature selection

**Figure 2:**
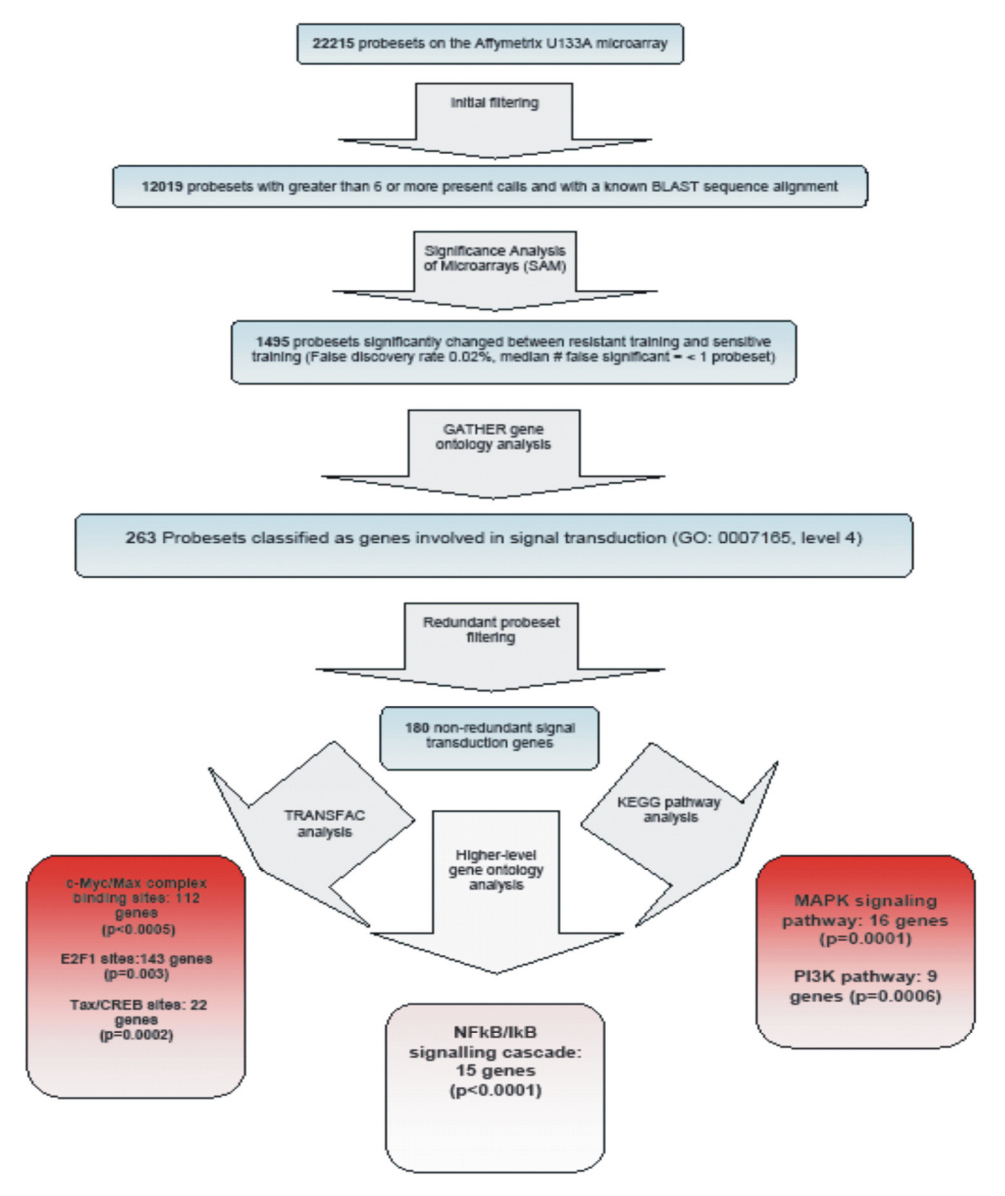
Feature selection and bioinformatics analysis for the 180 gene signature.

Based on the observation that cancer cell lines and tumors are selectively susceptible to inhibition of the EGFR signalling pathway and that sensitivity may not be directly correlated to EGFR mutation or amplification in all cases, we sought to identify a gene expression signature that is predictive of EGFR TKI sensitivity. Using independent replicates of drug-resistant cell lines (n = 11) and drug-sensitive cell lines (n = 14), we generated gene expression data, and using both bioinformatics and statistical analyses identified a set of genes that predict sensitivity to EGFR TKI, outlined in Figure 2 [see Additional Files 1 and 2].

Specifically, gene expression data generated from Affymetrix U133A arrays was filtered based on present/absent calls and BLAST sequence alignment. The 12,019 remaining probe sets were analyzed by Significance Analysis for Microarrays (SAM), resulting in 1495 differentially-expressed genes between the two groups, with a very low false discovery rate (0.025%) [24]. We wished to focus on genes found primarily to function in signalling transduction in order to minimize noise from genes that are less likely to be responsible for differences in EGFR TKI sensitivity. To accomplish this, we annotated the list of 1495 differentially expressed genes using GATHER, a web-based gene ontology algorithm which detects enrichment of GO terms at all levels within a submitted list of genes [42]. In the GATHER algorithm, p-values represent the probability of the term being similarly enriched in a randomly generated list of genes of identical size. A number of GO terms were significantly enriched within the 1,495 gene list, including signal transduction (GO:0007165, level 4, p < 0.0001), G-protein coupled receptor protein signalling pathway (GO:0007186, level 6, p < 0.0001) and cell surface receptor linked signal transduction (GO:0007166, level 5, p < 0.0001), consistent with the hypothesis that altered signalling cascades may represent a significant proportion of the variability in EGFR TKI response. We selected only those genes which were annotated under signal transduction (GO:0007165, level 4) to constitute a signature of EGFR sensitivity.

After GATHER annotation, 223 probesets remained, and several of these probesets were redundant with respect to their target gene. To minimize bias in subsequent analyses, we kept only the most significant of the redundant probesets. When all filtering steps were complete, we identified a 180-gene signal transduction-oriented expression signature of EGFR sensitivity (genes 1–50, Table 2, genes 51–180) [see Additional File 3]. The genes contained within the signature were re-annotated on higher levels of GO in to more precisely characterize the biologic roles of these genes that are differentially expressed in EGFR TKI sensitive cells. Using GATHER's GO pathway analysis, we found significant deregulation of the NFkB/IkB signalling cascade (15 genes, GO:0007249, level 7, p < 0.0001). Interestingly, KEGG pathway analysis of the 180-gene predictor revealed significant enrichment of pathways known act downstream of EGFR, including the MAPK signalling pathway (16 genes, p = 0.0001) and the phosphotidylinositol signaling pathway (9 genes, p = 0.0006) [see Additional File 4].

We also queried for significant enrichment of transcription factor binding sites among the 180-gene signature using TRANSFAC via GATHER. The genes clustered into three interesting and significant classes of DNA-binding domains: c-Myc/Max complex binding sites (112 genes, p < 0.0005), E2F1 sites (143 genes, p = 0.003) and Tax/CREB sites (22 genes p = 0.0002) [see Additional File 5].

### Internal and external validation using diagonal linear discriminant analysis

Diagonal linear discriminant analysis (DLDA) was performed on the 180-gene signature of EGFR sensitivity because this methodology performs well in classification problems concerning gene expression data [25]. For each unknown subject, DLDA calculates the distance of the unknown to average subject in each group of the training set with respect to the common diagonal covariance matrix. The unknown is then classified into the closest group.

The model was trained using the H1650 (n = 6), PC-9 (n = 5), and H3255 (n = 3) cell line samples as the sensitive group and the UKY-29 (n = 3) and A549 (n = 8) samples as the resistant group. The replicate measurements from each cell line were treated as independent samples by the subsequent algorithms to identify differentially expressed genes and build the discriminatory training model. We tested multiple predictive models, including the 10 and 50 most significantly deregulated genes (Table 2) of the 180-gene signature to determine the robustness of the predictor.

We performed a leave-one-out cross validation of the DLDA function. We assumed that one chip in the training set was an unknown, then performed the complete analysis based on the remaining chips, beginning with the initial filtering steps. This was performed for each chip of the initial training set in turn. Specifically, each time a chip was removed from the training set the following steps were performed; presence/absence call filtering, SAM analysis on the newly filtered data set, with the same delta-threshold used in the complete analysis training set, gene ontology filtering, redundant probesets were removed, the diagonal linear discriminate function was fit from the remaining 24 chips, and then EGFR TKI sensitivity of the removed chip was predicted based on the newly fit diagonal linear discriminate function. This was performed using the top 10 and 50 genes in each iteration, as well as the full gene list (range: 171–208 genes). Leave-one-out cross validation yielded a 0% misclassification rate. Likewise, we also performed a leave-a-group-out cross-validation in which an entire cell line set was removed and the model was iteratively rebuilt. This approach resulted in correct predictions for PC-9, H3255, UKY29, and H1650 samples but incorrectly classified 3 of the 8 replicates of A549 (88% accuracy) (data not shown).

To address the potential for bias due to unequal replicates used in the 180-gene model, a second predictive model of EGFR TKI sensitivity was trained using equal numbers of training data: the resistant group contains cell lines H460, A549, and UKY29 while the sensitive group contains cell lines H3255, PC-9, and H1650 using three replicates measurements for each line. The new model contains a 169-gene signature, 111 of these genes are in common with the 180-gene signature. The 10-, 50- and 169-gene models predict the validation cell lines identically and the tumor samples similarly as the 10-, 50-, and 180-gene models, with the exception of the A431 cell line in the 10-gene model [see Additional File 6]. That said, we will continue to use the 180-gene signature as it allows us the statistical power of all of our training data in the construction of the predictive model of EGFR TKI sensitivity.

The 180-gene models were then externally validated using a set of cell lines not used in training the model of EGFR TKI sensitivity. The characteristics of the cell lines included for external validation are found in Table 1. The K562 line was chosen as a negative control as it is a cancer cell line dependent on BCR-Abl expression to test if our predictor was, in fact, recognizing non-specific dependence on any activated kinase. The 10-, 50-, and 180-gene models were used to classify all cell lines. The models classified all samples correctly, with the exception of the UKY-29 sample in the 10-gene model and the H1975 cell line in all 3 models (see discussion). Additionally, we compared our genomic predictor (gene signatures and DLDA) to predictions based on mutational status alone, assuming sensitivity in the presence of exon 19 or 21 mutations, or resistance in the absence of EGFR mutations, or presence of an exon 20 mutation. Results are shown in Table 3. To assist the reader in reproducing the DLDA analysis described above, a Sweave script has been included [see Additional File 7].

To further assess the predictive accuracy of our model, we analyzed a NSCLC cell line dataset assayed on Affymetrix U133A microarrays from Girard and colleagues (GEO#GSE4824). These data include 14 lung cancer cell lines, of varied histologies, which were not included in our training model-Calu.3, H1299, H157, H1648, H2009, H2126, H820, HCC15, HCC2279, HCC4006, HCC44, HCC78, HCC827, and HCC95. Because NSCLC cell lines have a broad range of sensitivity to EGFR TKI, we chose an IC_50 _threshold of 2 μM to EGFR TKI as determined in Bunn et al for these 14 cell lines. Our genomic model of EGFR TKI sensitivity correctly classified 64% of the lines [26]. Increasing the threshold to 3 μM adds an additional correctly predicted sample (71%).

### Independent external validation on resected lung adenocarcinomas

**Figure 3:**
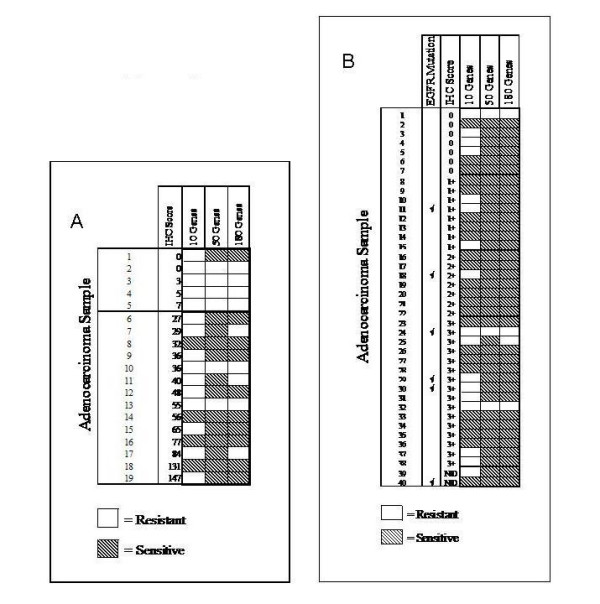
Classification of two independent collections of resected adenocarcinomas. Panel A: Tumors samples banked at H. Lee Moffitt Cancer Center and Research Institute were used for extraction of total RNA for probe preparation and hybridized to U133A arrays. IHC scoring was performed as previously described [27]. Thatched boxes represent predictions of sensitivity. Panel B: Tumors samples banked at Duke University were used for extraction of total RNA for probe preparation and hybridized to U133 2.0 arrays. pEGFR scoring is reported on a 4 point scale (0-3+). The presence of activating mutations within EGFR is also reported. Sensitive predictions are represented by a thatched box.

Given the accurate classification of the cell line data, we hypothesized that the signature of EGFR sensitivity should correctly classify resected tumors, and would result in appropriate predictions of response to EGFR TKIs *in vivo*. Two collections of resected adenocarcinomas, previously subjected to microarray analysis, were used to validate the predictive models of 10, 50, and 180 genes. Tumor samples obtained from H. Lee Moffitt Cancer Center and Research Institute (Tampa, Fl) were used for hybridization to Affymetrix U133A arrays and assayed by immunohistochemical (IHC) methods, scoring for phosphorylated EGFR (pEGFR) as previously reported [27]. Since persistently activated EGFR (pEGFR) may reflect underlying tumor reliance on EGFR and therefore sensitivity to EGFR TKI [27], we explored the relationship between classification by DLDA and pEGFR staining. Of the 19 tumors, 5 were either pEGFR-negative or exhibited very low pEGFR signal (<10 on a scale of 0–300) by IHC staining, while the remaining 14 stained with higher intensity of pEGFR. When the Moffitt tumors were predicted by DLDA, 4, 13, and 10 tumors classified as sensitive in the 10-, 50-, and 180-gene predictors respectively. Of the tumors that classified as sensitive, 100%, 92%, and 90%, respectively displayed higher degrees of pEGFR staining (>10). Of those tumors that were predicted to be resistant, 33%, 66% and 44% exhibited low levels or no pEGFR staining (<10), respectively [see Additional File 8]. Tumor classification for the three models as well as IHC scoring is presented in Figure 3, panel A.

Because mutational status of EGFR has been shown in select studies to correlate with tumor response to erlotinib and gefitinib [15,28], we chose to further validate our model on a series of resected adenocarcinomas for which mutational status, as well as pEGFR status was known. Adenocarcinomas from the Duke lung cancer cohort were used for hybridization to Affymetrix U133 Plus 2.0 arrays, and these data were imported into our models [29]. We were able to make predictions of EGFR TKI sensitivity of the Duke tumors using DLDA. We found that the 10-, 50- and 180-gene predictors identified 1/6 (17%), 5/6 (83%), and 5/6 (83%) of the tumors with EGFR mutations as sensitive, respectively. Of those tumors which classified as sensitive in the 10-, 50-, and 180-gene models, 82%, 78%, and 77% displayed positive staining for pEGFR, respectively. Of those tumors which were classified as resistant in the 10-, 50-, and 180- gene models, 24%, 33%, and 25% displayed no pEGFR staining, respectively. Classification of the data are shown for the Duke lung adenocarcinoma dataset in Figure 3, panel B.

## Discussion

The EGFR TKI erlotinib was shown to result in increased survival in previous clinical trials when used as monotherapy in previously treated patients with advanced NSCLC [30]. Toxicity to erlotinib is markedly lower than many alternative pharmacologic treatments, and would clearly be a preferred therapeutic option if survival was shown to be equivalent or better than treatment with other second line agents. Since only a fraction of patients respond to such therapy, *a priori *identification of responders could have a vast effect on survival. Many clinical parameters which have been shown to correlate with response to EGFR TKIs, including smoking history, gender, ethnicity, and tumor histology. Additionally, EGFR expression levels, phosphorylation status of EGFR, and mutations within the kinase domain [22,28,31] also correlate with sensitivity to some degree. While each of these predictors of response result in some overlap, potential responders to EGFR targeted therapeutics may be overlooked. In the same vain, a significant number of patients selected for treatment with EGFR TKI will fail therapy. Therefore, we undertook this study with the hypothesis that a gene expression signature of response will capture more of the variability within the tumor and improve prediction of EGFR TKI sensitivity than currently preferred methods. Furthermore, closer examination of the genes within this signature will allow for greater understanding of the effects of aberrant EGFR signalling, as well as potential elucidation of new drug targets.

Using NSCLC cell lines as tumor surrogates and previous findings as guidance, we sought to train our model by stratifying cell lines by drug sensitivity. Three sensitive cell lines were chosen for training data: H3255, PC9, and H1650. A549 cell line and UKY-29 cell lines were resistant to treatment and used for training data. The cell lines resistant to EGFR TKI harbour K-Ras mutations while the sensitive cell lines used in the training set all harbour EGFR mutations, as previously reported, and this finding is consistent with the hypothesis that K-Ras mutations and EGFR mutations are mutually exclusive in NSCLC [21].

Our hypothesis is anchored in the concept that while many factors correlate with sensitivity to EGFR inhibition, distinct combinations of signalling pathway deregulation may underlie the observed phenotype. Therefore, a gene expression signature capturing this complexity may be a more accurate predictor of response to EGFR TKI, and we defined a gene expression signature that utilizes our knowledge of signal transduction to model the phenotype of sensitivity.

Approximately 1500 genes were significantly different between our sensitive and resistant training cell lines, and while many of these genes may be important in our phenotype of response, we reasoned that a significant portion may be artifacts of two-dimensional growth and cell culture conditions. We filtered the 1500 differentially-expressed genes based on ontological annotation, allowing us to focus our signature on those genes which are important for cell signalling and are more likely to influence response to inhibition of the EGFR signalling cascade. To our knowledge, this is a novel approach to feature selection within a predictive gene signature study. A limitation of this approach is that genes which may contribute to pharmacokinetic variability such as transporters and metabolic enzymes would be omitted from the signature. Furthermore, markers of epithelial to mesenchymal transition (EMT), which have been shown to correlate with sensitivity to EGFR TKI [12,13] are not present in our final predictive signature due to the filtering by gene ontology. It is of note that the SAM analysis identified several EMT genes as differentially expressed within the 1500-gene training data set, such as vimentin, E-cadherin, and β-catenin (data not shown).

We defined a set of 180 features which represent differentially expressed genes that exhibit enrichment in signal transduction functions between EGFR-inhibition sensitive and EGFR inhibition-resistant cell lines, including a number of previously identified oncogenes such as Src, B-Raf, and PI3K that function downstream of EGFR activation. EGFR itself was identified as significantly deregulated and is consistent with the observation that EGFR expression may correlate with sensitivity [32].

GATHER allowed us to interrogate KEGG pathways in analysis of the genes included in the 180-gene signature and identified deregulation within the PI3K and MAPK pathways between sensitive and resistant cell lines. Interestingly, both of these pathways are downstream of EGFR, providing further evidence of their importance in NSCLC. Consistent with this finding, several subunits of PI3K were found highly-expressed in the EGFR TKI sensitive cells, including both the catalytic and regulatory subunits.

Analysis of transcription factor binding elements using GATHER also identified strong commonalities among the genes included in the signature. The high proportion of the genes are likely regulated by the E2F-family of transcription factors and/or c-MYC/MAX transcription factors suggesting common regulatory mechanisms may lead in to the phenotypic difference of EGFR TKI-sensitive and -resistant cells. Importantly, both activating E2Fs and Myc are recognized as essential cell cycle regulators and bind to promoters of genes important for driving cellular proliferation [33].

Many of the 180 features of our EGFR signature represent genes, described above, that were observed to have large differences with low variability in our system. Since our leave-one-out cross-validation yielded a 0% misclassification error, there may be concern that over-fitting of the model has occurred. A full leave-one-out cross validation (i.e. features are reselected and model parameters are rebuilt at each iteration) is a stringent and relatively unbiased estimate of the model building algorithm error [34,35]. However, to ensure that the treatment of replicate cell line samples as independent samples in our model did not result in cross-validation bias, we performed additional internal validation experiments. Subsequent cross-validation was performed in which the entire data from each cell line was removed (features were re-selected and weights were recalculated based on the data from only 4 cell lines, and the samples from the 5^th ^cell line were predicted using the new model). This method of cross-validation yielded a high degree of accuracy as well in that all cell lines predicted correctly, with the exception of 3 of 8 A549 samples (data not shown). We also constructed a second predictive model of EGFR TKI sensitivity using balanced numbers of replicates in both training classes. We found that although 111 genes of the resulting 169-gene model were common to the 180-gene signature the resulting model did not exactly replicate the classifications of the 10-, 50-, and 180-gene models. The differences could be due to a lack of statistical power in the second model or by utilizing all of the replicate measurements for the training cell lines. Thus, we may observe an artificial increase in our statistical power by using the 180-gene predictive model of EGFR TKI sensitivity.

We assessed the ability of this model to predict additional sets of gene expression data. To independently validate the signature, we used DLDA to classify cell lines that were not included in training the models. Additionally, we assessed the variability in predictive strength using multiple models. We found that predictions based on the most statistically significant 10 or 50 genes were similar to those made with the full data set. However, 10-gene model resulted in misclassification of both the UKY-29 and H1975 samples. This finding underscores the importance of including enough features in the model to account for variability found in the biological system of interest, a lung adenocarcinoma. Interestingly, the H1975 sample is seemingly misclassified in the 50- and 180-gene models as well, as this cell line harbours a second mutation in exon 20 that has been shown to confer resistance to the EGFR TKI gefitinib and erlotinib [23]. Importantly, however, recent reports have shown that the irreversible inhibitors of EGFR such as CL-387, 785 overcome this resistance [36]. Therefore, the double-mutant H1975 cell line, although insensitive to gefitinib and erlotinib, retains reliance on EGFR signalling pathways, providing an explanation for its classification using our models [37]. Furthermore, when compared to predictions based on mutational status alone, the genomic predictors (50- and 180-gene models) perform better in determining *a priori *sensitivity (Table 3).

We carefully selected the cell lines used as a validation set to ensure that our model was predictive of EGFR TKI sensitivity and not mutational status alone. The H358 adenocarcinoma cell line harbours a K-Ras mutant and no EGFR mutations, yet our predictor and data of others [13] identify this cell line as sensitive to EGFR inhibition. Furthermore, the A431 cell line was not derived from a lung adenocarcinoma, has both wildtype EGFR and K-Ras alleles, and is exquisitely sensitive to EGFR inhibition. However, K562 cell line is derived from a CML blast crisis patient, is wild-type for both EGFR and K-Ras, and is highly resistant to EGFR TKI. All three of these cell lines classify correctly and consistently among the 10-, 50-, and 180-gene predictors.

To strengthen confidence in our 180-gene model, we tested an independently derived set of NSCLC cell line microarray data that thus far is unpublished (Girard, GEO # GSE4824). Our signature correctly classified 64–71% of the cell lines, depending on IC_50 _threshold selection of resistance to EGFR TKI as determined in Bunn et al [26]. Of the four cell lines from the Girard set that were incorrectly predicted using our model, two were not of adenocarcinoma origin-H1299 (large cell carcinoma) and H157 (squamous cell carcinoma). Our predictor of sensitivity was trained using cell lines of adenocarcinoma origin and may then be more accurate when using similar data. That said, utilizing additional training data from cell lines of varied NSCLC histologies will likely improve the model for clinical use.

Finally, we assessed the ability of the predictive models to classify lung adenocarcinoma tumors. In the absence of clinical outcome or survival data from a prospective trial, we identified two datasets to which reasonable proxies for EGFR signalling and TKI sensitivity were available. These data included a set of 19 adenocarcinomas for which phosphorylated EGFR (pEGFR) was assessed using IHC and a set of 40 adenocarcinomas for which both pEGFR and EGFR mutational status was assessed. Classification based on 50 or 180 genes remained relatively constant demonstrating robust predictive power. Furthermore, classification of the tumors using 50- and 180-genes models identify a majority of the pEGFR positive samples in both datasets, as well as capturing 5 of 6 EGFR mutants in the Duke tumor dataset.

We identified several tumors in both the Moffitt and Duke datasets that demonstrate no detectable expression of pEGFR but classify as EGFR TKI sensitive using the predictive gene expression model. It is possible that IHC analysis is less sensitive than classification using the gene expression profile and is also dependent on sections stained and phospho-specific antibody used. That said, the tumors harbouring low levels of pEGFR predicted to be sensitive to EGFR TKI might possess deregulation of parallel signalling pathways that result in a gene expression phenotype that closely resembles activation of EGFR, and accordingly, these patients classify as sensitive to EGFR TKI.

We classified 83% (5/6) of the Duke cohort that were EGFR mutants as sensitive to EGFR by gene expression signature. While the predictor seems to have misclassified one tumor that harbors mutant EGFR, we note that others have reported that cell lines with activating EGFR mutations are also insensitive to EGFR TKI, and our predictive models may have identified a tumor that will not respond to treatment [10]. Additionally, in non- Japanese populations screened by EGFR mutational status prior to treatment with gefitinib, the response rate among those patients with either deletion or point mutation of EGFR was found to be 75% suggesting that mutation of EGFR is not sufficient for EGFR TKI sensitivity [38]. Thus, our tumor classifications accommodate the proportion of responders found in previous studies and while our approach may exceed those findings, future validation depends on comparing classification to response in a clinical study.

Because we did not have the EGFR TKI response data for the Moffitt and Duke tumor specimens, we used pEGFR staining and mutation status as surrogates for EGFR signalling, as described above. Combining both of the tumor data sets, our predictor of EGFR TKI sensitivity suggests that 80% of the tumors may be sensitive. Previous studies found that nearly 50% of patients with advanced stage IV NSCLC who had previously received cytotoxic chemotherapy had clinical benefit with EGFR TKI defined as either overt tumor response (shrinkage) or stable disease [1]. Since all the Moffitt and Duke tumors were of adenocarcinoma histology, a known clinical predictor of benefit to EGFR TKI, it is possible that the genomic predictor may accurately classify sensitivity in this group of tumors. It is also unclear the difference in EGFR TKI sensitivity between early stage lung cancers and widely metastatic cancers that have previously received cytotoxic chemotherapy. Studies are underway that address the sensitivity of early stage lung cancers to EGFR TKI. True assessment of the accuracy of our gene expression profiles to predict sensitivity of lung cancers to EGFR TKI will require prospective testing in patients.

## Conclusion

The gene expression signature of EGFR TKI sensitivity exhibits strong biological relevance as it encompasses many members of the EGFR signalling cascade. The prediction of sensitivity to EGFR inhibitors using DLDA models was accurate and robust within the cell line data. Furthermore, the DLDA predictive models suggest improved prediction of EGFR TKI sensitivity of human lung adenocarcinomas compared to single biomarkers alone. Clearly the next step in assessing the ability of this signature to improve upon existing methods must be determined in a clinical trial. We anticipate that use of gene expression predictors could advance patient-targeted therapy in this area.

## Methods

### Cell Culture

A549 cells were grown in RPMI 1640 (Invitrogen) with 2 mM L-glutamine containing 10% fetal bovine serum (FBS) (BioWest), 1.5 g/L sodium bicarbonate, 4.5 g/L glucose, 10 mM HEPES, and 1mM sodium pyruvate (Whittaker). H460 and UKY-29 cell lines [39] were generous gifts from Dr. Val Adams and Dr. John Yannelli, respectively, (University of Kentucky) and grown in DMEM (Invitrogen) + 10% FBS. H3255 cells were a gift from Dr. Frederick Kaye (NCI/Naval Medical Oncology, Betheseda, MD) and were grown in ACL4 media as described previously [5]. K562 cells were a gift from Dr. Rina Plattner (University of Kentucky) and were cultured in suspension in RPMI 1640 and 10% FBS. Human cancer cell lines H1650, H1975, PC9, H358 and A431 and grown as described [20,27].

### Cell line RNA isolation and Microarray Analysis

Cells were grown to subconfluence and passaged every three days. On the second day after passage, cell were harvested from a 150 mm plate and lysed in Trizol (Invitrogen). Total RNA was isolated and used for probe generation and hybridization to Affymetrix U133A DNA microarrays. Signal intensity values generated from Affymetrix MAS v5.0 software was used for statistical analysis, described below. Independent replicates of A549 (n = 8), UKY-29 (n = 3), H3255 (n = 3), PC-9 (n = 5), and H1650 (n = 6) were generated by using sequential passages of the cell populations. These replicates were treated as independent samples by the subsequent algorithms to identify differentially expressed genes and build the discriminatory training model. One replicate of each A431, H358, H460, H1975, and K562 was used for validation of the training model and a single replicate of each of the training lines omitted from the original models. The microarray data are available on maduk.uky.edu [see Additional File 1].

### Tumor Acquisition and Microarray Analysis

Duke cohort: After appropriate informed consent and Duke IRB approval, the analysis used an initial cohort 91 tumor samples obtained from patients with early stage (Ia/Ib, IIa/IIb and IIIa) NSCLC. From the resected lung specimens, percentage tumor content and histologic type of each tumor was ascertained before RNA extraction. Of the 91 RNA samples, 89 were of sufficient quality for gene expression analysis. Of the 89 samples, 40 were clearly identified as adenocarcinoma. Gene expression data was generated using an Affymetrix U133 2.0 plus array and processed as described previously [29]. The Affymettrix data for these samples is deposited on GEO under accession number GSE3141. EGFR mutational status (for exon 19 deletion and L858R) was determined using previously described techniques [4].

Moffitt cohort: Patients undergoing surgical resection of adenocarcinoma of the lung were consented to have tumor tissue stored and banked through a University of South Florida IRB approved protocol. Processing of the samples was performed as previously described [40]. The microarray data for the 180 probe sets used for classification are available [see Additional File 8].

### DNA Content Analysis

Cell lines were plated to 6 cm dishes in 10% media. Cells were starved in 0.5% media for 24 hours before treatment with 1 μM erlotinib (provided by Genentech, South San Francisco, CA) or DMSO for 72 hours. Floating and adherent cells were collected by trypsinization and centrifugation. Cell pellets were washed in 1 × phosphate-buffered saline (PBS) and fixed in 70% ice-cold ethanol. Pellets were washed in 1 × PBS,1% bovine serum albumin (BSA), and resuspended in 1 × PBS, 1% BSA, 50 ug/ml propidium iodide (Roche), and 0.5 mg/ml RNase A (Sigma) at 4°C. Cells were sorted by fluorescence activated cell sorting (FACS) (University of Kentucky core facility). Data was analyzed using ModFit LT (Verity Software, Topsham, ME). Apoptosis was recorded as the integrated sub-G_1 _peak.

### K-Ras and EGFR Sequencing

Actively growing cells were scraped into 1 × phosphate buffered saline (PBS) and pelleted by brief centrifugation. The cell pellets were lysed in 100 mM Tris HCl, pH 8.5; 5 mM EDTA; 0.2% SDS; 200 mM NaCl and 100 ug/mg proteinase K in a 500 ul volume at 55°C for several hours and the debris was pelleted by high speed centrifugation. Genomic DNA was precipitated from the supernatant, and the nucleic acid pellet was resuspended in 10 mM Tris-HCl, 1 mM EDTA. K-Ras exons 1 and 2 and EGFR exons 18–21 were independently sequenced as previously described [23,41].

### Gene Selection

EGFR TKI-sensitive and resistant cell line expression data was filtered to remove probesets with less than 6 'present' calls (<1/2 smallest n) between groups. Probesets with no single unique sequence by BLAST alignment were removed from the list [43]. The remaining genes were compared using Significance Analysis for Microarrays (SAM) [24]. Those genes which were determined to be differentially expressed between sensitive and resistant cell lines were annotated using GATHER [42,43]; and only those genes which annotated to signal transduction at level 4 (GO:0007165) were included in the discriminant analysis. Duplicate genes (i.e. different probesets which annotate to the same gene) were filtered by removing the least significant probeset(s) as determined by a 2-sample, equal variance t-test. The method of gene selection is described elsewhere [see Additional File 2].

### Diagonal Linear Discriminant Analysis

The genes in the final dataset were ordered by p-value in a two sample equal variance t-test. Diagonal linear discriminant analysis (DLDA) was performed using the top 10, top 50, and the complete gene signature (180 genes) in order to assess the stability and robustness of the model. A leave-one-out cross validation and external validation was performed on additional cell lines and adenocarcinomas. Adenocarcinomas hybridized to U133 Plus 2.0 arrays were filtered to remove genes not present on the U133A chip and mean chip intensities were standardized to the complete training data set. A Sweave script is included that carries out the DLDA analysis [see Additional File 7].

## Authors' contributions

J.B. and C.S. were responsible for generating gene expression data and constructing and validating the resulting predictive models. J.B. co-authored the manuscript. A.P. provided the Duke tumor samples and offered helpful suggestions. E.H. provided the Moffitt tumor samples and was instrumental in development of the project. A.S. provided statistical expertise. E.P.B. co-authored the manuscript and was responsible for project development. All contributing authors reviewed and approved the final copy of this manuscript.

## Supplementary Material

Additional File 1 Inventory of Affymetrix U133A microarray data as available on  All training and validation sets are listed. At the MADUK home page, choose Public Login and 'PENNIB' as the experimenter to access all Affymetrix files. Click here for file

Additional File 2 Sheet 1: Probesets excluded from the analysis because they did not align to a single transcript in a BLAST alignment analysis, as determined by Girard et al, manuscript in preparation.
Sheet 2: SAM output, with parameters for analysis. These were probesets which were included in the subsequent GO analyses to determine deregulated signal transduction genes. Click here for file

Additional File 3 **Genes 51–180 of the gene signature of EGFR TKI sensitivity**. For each gene, the Affymetrix probe ID, gene name, gene description, and p-value are given. Click here for file

Additional File 4 **KEGG Pathway analysis of the 180 gene signature via GATHER**. Genes contained under each significant pathway map are given. Click here for file

Additional File 5 **TRANSFAC analysis of the 180 gene signature via GATHER**. Significant transcription factor binding sites and genes containing them are given. Click here for file

Additional File 6 Diagonal linear discriminant analysis of NSCLC cell lines using an equally balanced predictor. Click here for file

Additional File 7 Sweave scripts (.TEX and .RNW), PDF file describing contents of Sweave script, and .TXT files (training and validation data). Click here for file

Additional File 8 Affymetrix U133A microarray data for the 180 probesets used for the DLDA models to classify the Moffitt tumors. MAS v5.0 values and present/absent calls are available for each tumor. Click here for file

## Figures and Tables

**Table 1:** Characterization of cell lines used in training and validation

					Affymetrix U133A chips		
						
	Cell Line	Type	Sensitivity to EGFR TKI	K-Ras Status	(n) in training	(n) in validation	EGFR Status
Training	A549	AC	No	Mutant (Codon 12)	8	N/A	Wt ^1^
	UKY-29	AC	No	Mutant (Codon 61)^1^	3	N/A	Wt ^1^
	H1650	AC	Yes	Wt	6	N/A	Mutant (DelE746-A750)^1^
	PC-9	AC	Yes	Wt	5	N/A	Mutant (DelE746-A750)^1^
	H3255	AC	Yes	Wt	3	N/A	Mutant (L858R) ^1^

Validation	H358	AC	Yes	Mutant (Codon 12)	0	1	Wt^1^
	H460	Large Cell	No	Mutant (Codon 61)	0	1	Wt^1^
	H1975	AC	No	Wt	0	1	Mutant (L858R, T790M) ^1^
	K562	CML	No	Wt	0	1	Wt^1^
	A431	Epidermoid	Yes	Wt	0	1	Wt (Amplified)^1^

^1 ^Assayed in this study

AC: Adenocarcinoma

CML: Chronic Myelogenous Leukemia

**Table 2:** Genes 1–50 of the 180-gene signature of EGFR TKI sensitivity

Probeset	Gene	Description	p-value
205891_at	ADORA2B	adenosine A2b receptor	1.65347E-12
213434_at	EPIM	epimorphin	2.03526E-12
211475_s_at	BAG1	BCL2-associated athanogene	1.2089E-11
201716_at	SNX1	sorting nexin 1	1.3942E-11
219933_at	GLRX2	glutaredoxin 2	2.82157E-11
204513_s_at	ELMO1	engulfment and cell motility 1	2.92588E-11
203011_at	IMPA1	inositol(myo)-1(or 4)-monophosphatase 1	4.20475E-11
202743_at	PIK3R3	phosphoinositide-3-kinase, regulatory subunit 3 (p55, gamma)	4.51605E-11
204491_at	PDE4D	Phosphodiesterase 4D, cAMP-specific	8.05036E-11
204000_at	GNB5	guanine nucleotide binding protein (G protein), beta 5	8.7681E-11
204115_at	GNG11	guanine nucleotide binding protein (G protein), gamma 11	1.02678E-10
218913_s_at	GMIP	GEM interacting protein	2.64411E-10
200994_at	IPO7	importin 7	2.65447E-10
202286_s_at	TACSTD2	tumor-associated calcium signal transducer 2	2.75325E-10
209035_at	MDK	midkine (neurite growth-promoting factor 2)	7.31553E-10
218995_s_at	EDN1	endothelin 1	7.75626E-10
219855_at	NUDT11	nudix (nucleoside diphosphate linked moiety X)-type motif 11	8.77697E-10
209678_s_at	PRKCI	protein kinase C, iota	1.04253E-09
202501_at	MAPRE2	microtubule-associated protein, RP/EB family, member 2	2.31343E-09
212117_at	RHOQ	ras homolog gene family, member Q	3.22134E-09
206277_at	P2RY2	purinergic receptor P2Y, G-protein coupled, 2	3.92313E-09
209295_at	TNFRSF10B	tumor necrosis factor receptor superfamily, member 10b	4.33798E-09
205376_at	INPP4B	inositol polyphosphate-4-phosphatase, type II, 105kDa	4.50987E-09
206722_s_at	EDG4	endothelial differentiation, lysophosphatidic acid GPCR,4	7.96715E-09
205673_s_at	ASB9	ankyrin repeat and SOCS box-containing 9	1.24878E-08
201471_s_at	SQSTM1	sequestosome 1	1.34231E-08
204352_at	TRAF5	TNF receptor-associated factor 5	1.46887E-08
206907_at	TNFSF9	tumor necrosis factor (ligand) superfamily, member 9	1.57771E-08
218150_at	ARL5	ADP-ribosylation factor-like 5	2.04888E-08
205459_s_at	NPAS2	neuronal PAS domain protein 2	2.22961E-08
205455_at	MST1R	macrophage stimulating 1 receptor (c-met-related tyrosine kinase)	2.45512E-08
202641_at	ARL3	ADP-ribosylation factor-like 3	2.78193E-08
201667_at	GJA1	gap junction protein, alpha 1, 43kDa (connexin 43)	2.86113E-08
210512_s_at	VEGF	vascular endothelial growth factor	2.90316E-08
212104_s_at	RBM9	RNA binding motif protein 9	5.42805E-08
200762_at	DPYSL2	dihydropyrimidinase-like 2	5.43168E-08
221235_s_at	TGFBRAP1	transforming growth factor, beta receptor associated protein 1	5.51367E-08
211302_s_at	PDE4B	phosphodiesterase 4B, cAMP-specific	5.51731E-08
205080_at	RARB	retinoic acid receptor, beta	7.03586E-08
202266_at	TTRAP	TRAF and TNF receptor associated protein	7.2889E-08
205240_at	GPSM2	G-protein signalling modulator 2 (AGS3-like, C. elegans)	8.30858E-08
213798_s_at	CAP1	CAP, adenylate cyclase-associated protein 1 (yeast)	8.61121E-08
221819_at	RAB35	RAB35, member RAS oncogene family	8.9216E-08
207011_s_at	PTK7	PTK7 protein tyrosine kinase 7	9.78716E-08
204255_s_at	VDR	vitamin D (1,25- dihydroxyvitamin D3) receptor	1.1087E-07
208864_s_at	TXN	thioredoxin	1.34274E-07
209885_at	RHOD	ras homolog gene family, member D	1.50021E-07
201923_at	PRDX4	peroxiredoxin 4	1.6148E-07
204392_at	CAMK1	calcium/calmodulin-dependent protein kinase I	2.24378E-07
203269_at	NSMAF	neutral sphingomyelinase (N-SMase) activation associated factor	2.59238E-07

* Genes 51–180 are included [see Additional File 3]

**Table 3:** Diagonal linear discriminant analysis of NSCLC cell lines

			Predicted sensitivity to EGFR TKI			
			
	Cell Line	Experimental Sensitivity to EGFR TKI (erlotinib)	Prediction based on analysis of mutational status alone (Exons 18–21)	Genomic signature/DLDA		
				
				10-genes	50-genes	180-genes
**Training**	A549	No	√	√*	√	√
	UKY-29	No	√		√	√
	H1650	Yes	√	√	√	√
	PC-9	Yes	√	√	√	√
	H3255	Yes	√	√	√	√

**Validation**	H358	Yes		√	√	√
	H460	No	√	√	√	√
	H1975	No	√			
	K562	No	√	√	√	√
	A431	Yes		√	√	√
		
		**% Correct**	80%	80%	90%	90%

Predictions of EGFR TKI sensitivity are denoted for ten cell lines used in training/validation. Column 2 demonstrates experimental sensitivity to an EGFR TKI, erlotinib (Table 1). Column 3 demonstrates prediction of sensitivity using mutational status of EGFR. Columns 4–6 denote prediction of sensitivity of the cell lines using the 10, 50, and 180 gene signatures in DLDA. √: denotes correct prediction based on experimental sensitivity to EGFR TKI. *: Leave-a-group-out cross-validation incorrectly predicts 3 of 8 replicates of this cell line.
