# Supplementary material for: Gene expression patterns that predict sensitivity to epidermal growth factor receptor tyrosine kinase inhibitors in lung cancer cell lines and human lung tumors
Source: BMC Genomics. 2006 Nov 10;7:289. doi: 10.1186/1471-2164-7-289 (PMC1660550; doi:10.1186/1471-2164-7-289)
Supplement: Additional File 3 — Genes 51–180 of the gene signature of EGFR TKI sensitivity. For each gene, the Affymetrix probe ID, gene name, gene description, and p-value are given. [file 1471-2164-7-289-S3.doc]

Additional File 3: Genes 51-180 of the EGFR TKI sensitivity expression signature Supplementary Table 1, continued

Supplementary Table 1, continued
