# Supplementary material for: Gene expression patterns that predict sensitivity to epidermal growth factor receptor tyrosine kinase inhibitors in lung cancer cell lines and human lung tumors
Source: BMC Genomics. 2006 Nov 10;7:289. doi: 10.1186/1471-2164-7-289 (PMC1660550; doi:10.1186/1471-2164-7-289)
Supplement: Additional File 5 — TRANSFAC analysis of the 180 gene signature via GATHER. Significant transcription factor binding sites and genes containing them are given. [file 1471-2164-7-289-S5.doc]

Additional File 5: TRANSFAC Annotations
