# Supplementary material for: Gene expression patterns that predict sensitivity to epidermal growth factor receptor tyrosine kinase inhibitors in lung cancer cell lines and human lung tumors
Source: BMC Genomics. 2006 Nov 10;7:289. doi: 10.1186/1471-2164-7-289 (PMC1660550; doi:10.1186/1471-2164-7-289)
Supplement: Additional File 6 — Diagonal linear discriminant analysis of NSCLC cell lines using an equally balanced predictor. [file 1471-2164-7-289-S6.doc]

## Additional File 6 – Diagonal linear discriminant analysis of NSCLC cell lines using an equally balanced predictor

|  | Cell Line | Experimental Sensitivity to EGFR TKI (erlotinib) | Predicted sensitivity to EGFR TKI | | | |
| --- | --- | --- | --- | --- | --- | --- |
|  |  |  | Prediction based on analysis of mutational status alone (Exons 18-21) | Genomic signature / DLDA | | |
|  |  |  |  | 10- genes | 50-genes | 169- genes |
| **Training Set** | A549 | No | √ | √* | √ | √ |
|  | UKY-29 | No | √ | √ | √ | √ |
|  | H460 | No | √ | √ | √ | √ |
|  | PC-9 | Yes | √ | √ | √ | √ |
|  | H3255 | Yes | √ | √ | √ | √ |
|  | H1650 | Yes | √ | √ | √ | √ |
| **Validation Set** | H358 | Yes |  | √ | √ | √ |
|  | H1975 | No | √ |  |  |  |
|  | K562 | No | √ | √ | √ | √ |
|  | A431 | Yes |  |  | √ | √ |
|  |  | **% Correct** | 80% | 80% | 90% | 90% |

Predictions of EGFR TKI sensitivity are denoted for ten cell lines used in training/validation. Column 2 demonstrates experimental sensitivity to an EGFR TKI, erlotinib. Column 3 demonstrates prediction of sensitivity using mutational status of EGFR. Columns 4-6 denote prediction of sensitivity of the cell lines using 10, 50, and all 169 genes in DLDA. √: denotes correct prediction based on experimental sensitivity to EGFR TKI. * Leave-a-group-out cross-validation incorrectly predicts this cell line.
