# Supplementary material for: Gene expression patterns that predict sensitivity to epidermal growth factor receptor tyrosine kinase inhibitors in lung cancer cell lines and human lung tumors
Source: BMC Genomics. 2006 Nov 10;7:289. doi: 10.1186/1471-2164-7-289 (PMC1660550; doi:10.1186/1471-2164-7-289)
Supplement: Additional File 7 — Sweave scripts (.TEX and .RNW), PDF file describing contents of Sweave script, and .TXT files (training and validation data). [file 1471-2164-7-289-S7.zip › DLDA-Sweave.pdf]

A Diagonal Linear Discriminant Analysis for Genomic Data

Justin Balko and Christopher Saunders

October 10, 2006

This Sweave report will guide you (the user) through the DLDA analysis performed in the manuscript 'Gene Expression Patterns that Predict Sensitivity to Epidermal Growth Factor Receptor Tyrosine Kinase Inhibitors in Lung Cancer Cell Lines and Human Lung Tumors' by Justin M. Balko, Anil Potti, Christopher Saunders, Arnold Stromberg, Eric B. Haura and Esther P. Black This report can be accurately reproduced by using the command "Sweave("DLDA-Sweave.rnw")" in R, which will produce the .tex file. This tex file is then processed by LaTEX to produce the PDF report

There are three functions which are included in the .rnw file which are necessary to perform the analysis:

- T-test function
- Scoring function
- Validation function

The code can be extracted by Stangle, but will not be echoed in this report.

The data to be analyzed must also be read in. The three data files should be named as they were included in the ZIP file:
("Validation.txt")
("S-training.txt")
("R-training.txt")

In order to perform additional analyses on a separate validation set, please see the Validation.txt file for formatting. The current code will require that the filename of the .txt file containing the new data remain "Validation.txt", else it will not be found by the program. Therefore, one can replace the sample data in Validation.txt with one's own data, or simply remove the supplied Validation.txt from the active directory and create a new file of similar format named "Validation.txt" containing user data in the active directory. For the analysis to work on additional validation datasets, all signature ProbesetIDs (Affymetrix U133A or U133 plus 2.0) must be included in the Validation.txt file, and they must be in the same order. Extraneous probesets (those that are not included in the 180-gene signature) need not be included in the Validation.txt file.
Note: Affymetrix 2.0 data may need to be scaled prior to analysis
The data is now read into Sweave:

```
> r.training = read.delim("R-training.txt")
> s.training = read.delim("S-training.txt")
> validation = read.delim("Validation.txt")
```

The tests are performed, using 180 genes (The full model), The 50 most significant genes, and the 10 most significant genes. Three tables and three figures are included in the report, one of each for the 10-, 50-, and 180-gene models. The plots depict the group scores. Group scores are measures of the distance of the unknown sample to the the training samples. A high group score means that there is a small distance between the unknown sample and the members of that group.

	S/R	S-Score	R-Score
H460.Val.	R	3.50712234649727	10.4153085868305
H1975.Val.	S	5.67206194078097	4.25318427966896
A431.Val.	S	9.90613049479836	5.15169601231139
H358.val.	S	11.3158589059136	5.83256206195803
K562.Val.	R	8.20864269776948	8.28824229624357
A549.1	R	0.554188203822184	9.28945878097809
A549.2	R	0.588872033254224	9.2360493763814
A549.3	R	0.557535724750725	8.4592292920568
A549.4	R	0.590159682229932	8.99171641266593
A549.5	R	1.08663682218889	7.6084752132606
A549.6	R	1.35373323191940	8.76861418313954
A549.7	R	0.79182266617047	7.91413810956302
A549.8	R	0.478082928313167	7.93254244372126
UKY.29.1	R	1.39587232127476	9.31098915385664
UKY.29.2	R	1.28523517086167	8.99906151415022
UKY.29.3	R	1.30619149539736	8.29757497624548
H1650.1	S	8.60140844896934	0.848568730677319
H1650.2	S	7.93059016092265	0.879545479104146
H1650.3	S	7.93391266011578	0.829919681845509
H1650.4	S	8.00465873901919	0.671528398873181
H1650.5	S	10.0417780508982	1.74306956183656
H1650.6	S	7.78981131800014	0.913770140406034
PC.9.1	S	7.9649810865547	0.86824376963868
PC.9.2	S	8.64317028614197	0.75604855627922
PC.9.3	S	9.16688622097646	0.851446779855058
PC.9.4	S	8.70580573956531	0.69511228819551
PC.9.5	S	8.74283331805926	0.787185640037256
H3255.1	S	9.00239358255087	1.08548760486508
H3255.2	S	9.18538545055371	0.977978042918286
H3255.3	S	9.25016997219033	1.10376504528538

Table 1: Predictions using 180-gene model

	S/R	S-Score	R-Score
H460.Val.	R	4.3384660323263	18.8672343653333
H1975.Val.	S	9.31553790172432	6.36945763079822
A431.Val.	S	19.4656161239126	9.65181402006183
H358.val.	S	22.0894740172941	11.2509241879786
K562.Val.	R	9.26267310277834	10.9427640910634
A549.1	R	0.615287632165346	17.4722839014836
A549.2	R	0.625013083904596	16.8497138473933
A549.3	R	0.662988133378457	15.0997804675151
A549.4	R	0.602357145391252	16.3232611992685
A549.5	R	0.991320094101613	12.3694333907967
A549.6	R	1.49716686366224	14.6316501104911
A549.7	R	0.753191355621748	14.1036193885591
A549.8	R	0.501444675638464	14.0666259905501
UKY.29.1	R	1.51780068800451	18.128070889321
UKY.29.2	R	1.35308649829935	17.9947596323082
UKY.29.3	R	1.51060268488827	15.6397684010632
H1650.1	S	15.5810851518594	0.777429133164602
H1650.2	S	14.8660661681941	0.766506465250738
H1650.3	S	15.1361631074745	0.743452391321581
H1650.4	S	16.2706089855095	0.497316290896076
H1650.5	S	16.5891015036662	1.61205171914070
H1650.6	S	15.0251387304060	1.02306955536312
PC.9.1	S	14.1379651264000	0.756428259183398
PC.9.2	S	14.8947754191373	0.667795883997997
PC.9.3	S	15.3931196645767	0.85071237268764
PC.9.4	S	14.6632471023698	0.584373740412371
PC.9.5	S	15.0363173795572	0.61631600448272
H3255.1	S	17.4612775847571	1.22673694386595
H3255.2	S	17.0783895660751	1.14411881728270
H3255.3	S	16.4802962996628	1.10343356789456

Table 2: Predictions using 50-gene model

	S/R	S-Score	R-Score
H460.Val.	R	4.60479666490942	24.2391403119443
H1975.Val.	S	26.8545796468766	11.1113881845657
A431.Val.	S	19.7476122357169	4.55735184936479
H358.val.	S	11.9641456380827	8.7820093937359
K562.Val.	R	5.99679865234377	14.9765915353630
A549.1	R	0.79811768591128	28.9174241094385
A549.2	R	0.407588833643069	26.616131381316
A549.3	R	1.53997929488633	25.8970722147442
A549.4	R	0.533446754777845	27.6421206103499
A549.5	R	1.87871265277492	18.1760426996498
A549.6	R	1.65798215459624	28.4399530839154
A549.7	R	0.623099614334213	25.5875588374383
A549.8	R	0.820031290342609	22.0751323435286
UKY.29.1	R	0.699381624965589	24.8552586557242
UKY.29.2	R	1.20006066034033	28.1917494200501
UKY.29.3	R	1.31778170965630	21.6775643270202
H1650.1	S	25.7521809790014	0.492799687831408
H1650.2	S	26.5298913981107	0.804788580354355
H1650.3	S	24.8986723873364	0.660037499598239
H1650.4	S	27.2748543384829	0.306593390149513
H1650.5	S	25.8360450829288	1.84285205485106
H1650.6	S	24.4587508718758	1.11081121817650
PC.9.1	S	26.0117890462651	1.01799862129357
PC.9.2	S	22.9842649993513	0.612066584964118
PC.9.3	S	19.7807742034792	0.434823362924049
PC.9.4	S	22.8064125163342	0.479761775173056
PC.9.5	S	24.9104932304091	0.441372598478779
H3255.1	S	26.6664159478554	1.01919705569701
H3255.2	S	27.3783065523207	1.07455418739362
H3255.3	S	25.5438348697701	1.22616110688599

Table 3: Predictions using 10-gene model

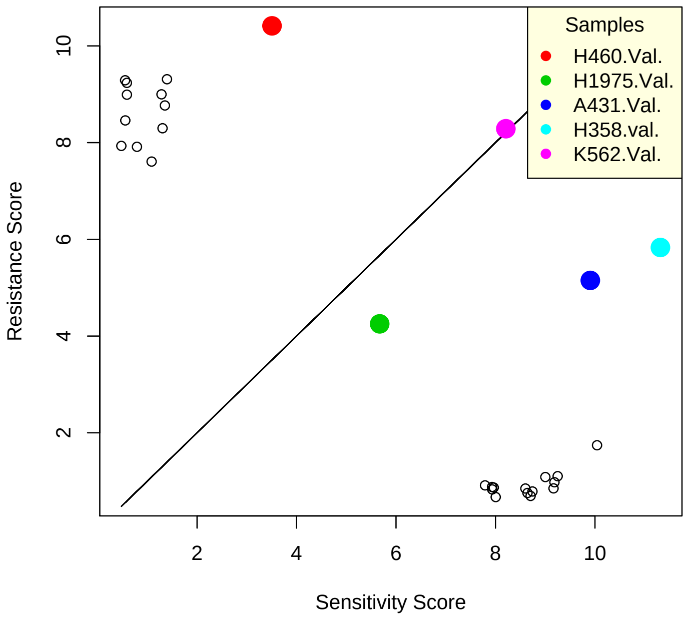

Figure 1: 180-gene prediction model

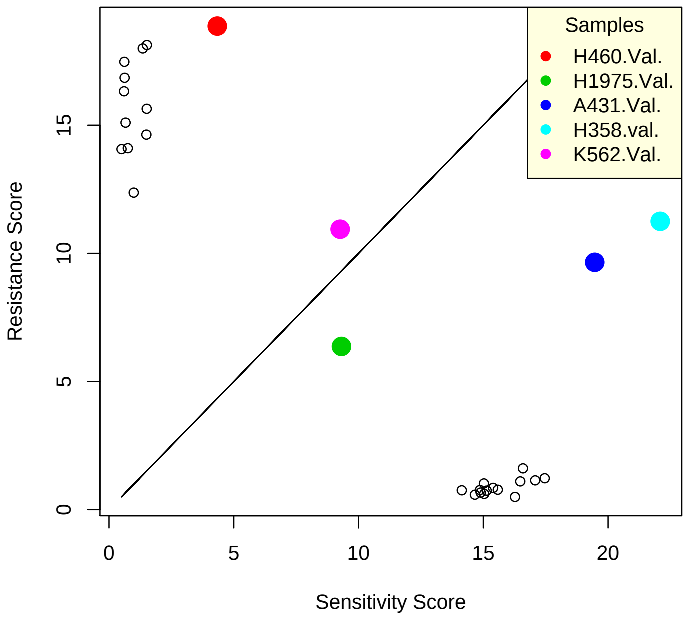

Figure 2: 50-gene prediction model

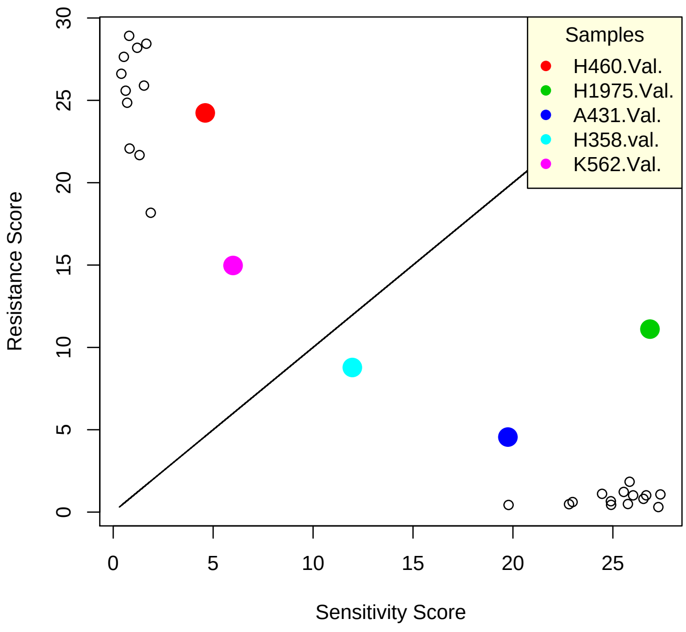

Figure 3: 10-gene prediction model
